# Integrating One Health governance in China: Assessing structural implementation and operational entry points

**DOI:** 10.1016/j.onehlt.2025.101209

**Published:** 2025-09-17

**Authors:** Qi-yu Zhang, Yan-Yan Zhang, Jing-Shu Liu, Xin-Chen Li, Ze-Lin Zhu, Xin-Yu Feng, Le-Fei Han, Chen-Di Zhu, Hein-Min Tun, Lian Zong, Xiaoxi Zhang

**Affiliations:** a School of Global Health, Chinese Center for Tropical Diseases Research, Shanghai Jiao Tong University School of Medicine, Shanghai, People's Republic of China; b Institute of One Health, Shanghai Jiao Tong University, Shanghai, People's Republic of China; c School of Public Health, Shanghai Jiao Tong University School of Medicine, Shanghai, People's Republic of China; d Shanghai Baoshan District Center for Disease Control and Prevention (Shanghai Baoshan District Health Inspection and Supervision Institute), Shanghai, People's Republic of China; e National Institute of Parasitic Diseases at Chinese Center for Disease Control and Prevention (Chinese Center for Tropical Diseases Research), NHC Key Laboratory of Parasite and Vector Biology, WHO Collaborating Centre for Tropical Diseases, Shanghai, People's Republic of China; f Jockey Club School of Public Health and Primary Care, Faculty of Medicine, The Chinese University of Hong Kong, Hong Kong, SAR 999077, China; g System Microbiology and Antimicrobial Resistance (SMART) Lab, Li Ka Shing Institute of Health Sciences, Faculty of Medicine, The Chinese University of Hong Kong, Hong Kong, SAR 999077, China

**Keywords:** One Health governance, One Health implementation, One Health entry points

## Abstract

**Background:**

In China, despite the establishment of a top-level One Health framework to address the interconnected risks of zoonotic diseases, antimicrobial resistance (AMR), and environmental degradation, significant challenges persist in translating this vision into effective implementation. The country's large agricultural sector, dense population, and diverse ecosystems require coordinated governance across human, animal, and environmental health sectors. However, gaps remain in resource allocation, cross-sector coordination, and policy integration. This study evaluates China's One Health governance through four structural domains—Monitoring & Evaluation, Intervention & Response, Surveillance & Early Warning, and Capacity Building—and four operational entry points—Technology, Information, Human Resources, and Finance. The aim is to identify strengths, gaps, and provide actionable recommendations for improvement.

**Methods:**

A qualitative approach was employed, involving 41 in-depth expert interviews, policy document analysis, and a systematic literature review. Interviews were conducted with stakeholders from public health, veterinary services, and environmental agencies, focusing on key domains of the One Health implementation. Data were thematically coded using NVivo 12.0 and analyzed using Grounded Theory. The results were cross-checked through intercoder reliability testing to provide actionable recommendations.

**Results:**

While regular reporting mechanisms exist for Monitoring & Evaluation across human, animal, and environmental health sectors, real-time assessments remain insufficient, limiting timely, multisectoral responses to emerging health risks. A tiered emergency system supports Intervention & Response, but inefficiencies in cross-sector resource allocation and coordination hinder its effectiveness. In Surveillance & Early Warning, fragmented data collection and interdepartmental silos across different health domains impede comprehensive and integrated risk monitoring. Despite existing Capacity Building programs, large-scale interdisciplinary exercises and continuous skill development opportunities remain scarce. Operationally, diagnostic technologies like polymerase chain reaction (PCR) and geographic information system (GIS) are available but unevenly distributed. Data-sharing mechanisms exist, but bureaucratic delays and inconsistent standards hinder integration. Workforce gaps, an aging cohort, and insufficient multidisciplinary training threaten the sustainability of the One Health framework. Operational budgets and personnel incentives remain inadequate, limiting the impact of financial investments.

**Conclusion:**

Despite progress in policy and infrastructure, China faces critical gaps in One Health implementation, particularly in real-time monitoring, cross-sectoral coordination, data integration, workforce capacity, and financial support. Addressing these gaps is crucial for enhancing One Health governance and offers valuable lessons for other regions with similar challenges in integrating human, animal, and environmental health.

## 1 Background

One Health, an integrated approach recognizing the interconnectedness of human, animal, and environmental health, has become increasingly critical in addressing global health challenges. While the theoretical framework behind One Health has gained traction globally, its practical implementation has remained a challenge, especially at the national level. Despite the concerted efforts of international organizations [1], there remains a notable gap in governance at the country level [2], particularly in countries like China, where integration of One Health principles into policy and practice has encountered significant obstacles.

China has undertaken efforts to mitigate health risks related to infectious diseases, zoonoses, and antimicrobial resistance (AMR). However, a comprehensive One Health governance structure remains underdeveloped. Fragmentation across sectors and the absence of a unified framework have limited the effectiveness of multisectoral coordination. These challenges are compounded by rapid urbanization and high-density human-animal-environment interactions. In response, global and national actors have emphasized the need to identify practical mechanisms for advancing One Health implementation. Prior studies have discussed various entry points—such as surveillance systems, AMR regulation, and environmental conservation—that offer potential for cross-sectoral integration [3]. These domains suggest that leveraging existing systems rather than creating entirely new mechanisms could be a more sustainable path toward improving One Health outcomes.

However, the lack of a comprehensive understanding of the existing governance structures, coupled with fragmented institutional support, means that the potential benefits of integration are not being fully realized. Frontline health workers—those on the ground in disease surveillance, AMR control, and environmental management—often have valuable insights into the practical challenges of implementing One Health. Yet, these insights are rarely captured in formal policy-making processes, which limits their ability to inform decision-making and improve governance.

To capture such insights, this study conducted field interviews in Jiangxi Province (including Nanchang, Yongxiu County, and Duchang County) and Hainan Province, supplemented by national-level expert interviews. Yongxiu County, in particular, represents a typical county-level locality in a schistosomiasis-endemic area near Poyang Lake, with a population relying heavily on agriculture and aquaculture. Its high exposure to zoonotic risks and recent One Health-related reforms—such as integrated evaluation mechanisms and cross-sectoral coordination pilots—make it a valuable illustrative case for examining the practical challenges and opportunities of One Health governance in China.

Given these contextual challenges, there is a need for systematic inquiry into the structural and operational components that shape the implementation of One Health in China. Particularly, understanding how key governance domains—such as monitoring, surveillance, emergency response, and workforce development—interact with operational enablers like technology, information systems, financing, and human resources is critical for identifying practical leverage points for integration.

This study therefore focuses on assessing China's One Health governance by examining four structural domains and four operational entry points, as conceptualized in recent frameworks. Drawing on empirical data from expert interviews, policy documents, and literature, it seeks to identify current strengths, gaps, and cross-sectoral coordination challenges that influence One Health implementation. The findings aim to inform policy design and offer actionable insights for strengthening integrated health governance in China and other contexts with similar multisectoral complexity.

## 2 Methods

### 2.1 Research overview

This study forms a focused component of a broader investigation into the One Health governance landscape in China. It builds directly on the foundational work of Li et al. [4], adopting the structured, phased research approach developed in that study. Following the methodology of Li et al., this study proceeds through three interrelated phases. First, it assesses the current implementation status of One Health initiatives in China and identifies potential entry points, establishing a baseline understanding of the practical landscape. Second, through literature review, policy analysis, and expert consultation, it identifies major challenges that hinder effective implementation and the operationalization of these entry points. Third, it synthesizes the findings to develop actionable recommendations aimed at strengthening governance, with a particular focus on optimizing implementation pathways and effectively leveraging identified entry points. Expert consensus is used to prioritize and refine these recommendations.

**Fig. 1:**
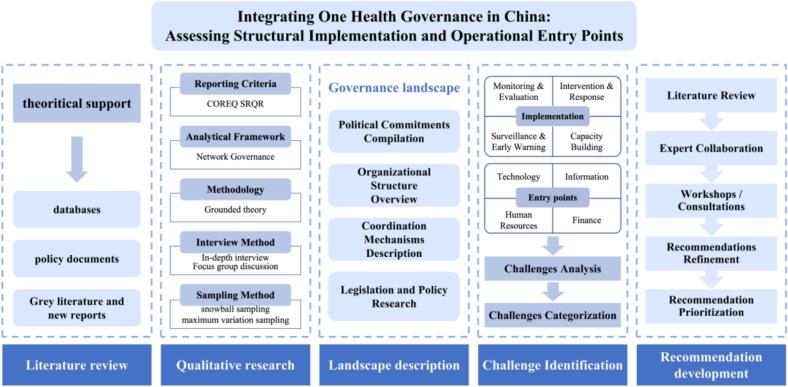
Flowchart.

Whereas Li et al. emphasized overall One Health strategy and governance mechanisms, this study shifts the analytical lens to practical implementation and the identification of context-specific entry points in China. Qualitative methods—including in-depth interviews and focus group discussions—are used to capture diverse perspectives. Fig. 1 presents the research framework and outlines the methods applied in each phase.

### 2.2 Literature review

A systematic literature search was conducted to identify peer-reviewed studies and policy documents related to One Health governance in China. English-language sources were retrieved from PubMed, ScienceDirect, Web of Science, and MEDLINE; Chinese-language sources were accessed via CNKI, Wanfang, and the China Biomedical Literature Service System. The search covered publications from 2001 to 2023 to capture the development of One Health discourse over time. The primary search strategy employed core terms related to One Health governance. To ensure comprehensive coverage, supplementary searches were conducted using keywords associated with zoonotic disease control. In addition to academic literature, relevant national laws, regulations, and official guidelines were obtained from the websites of key Chinese ministries and agencies. Grey literature—including technical reports, working papers, and conference proceedings—was reviewed, along with targeted searches for media reports using Google, Baidu, and Bing.

### 2.3 Study participants

This study employed purposive sampling to recruit key stakeholders engaged in One Health initiatives across multiple sectors in China. Participants were selected from three primary categories: (i) national, sub-national, and local level experts involved in One Health-related governance, policy development, or intersectoral coordination; (ii) technical personnel and frontline practitioners, to capture the implementation experiences; and (iii) professionals from human, animal, or environmental health sectors with relevant institutional affiliations. Eligibility criteria for participants included: (i) at least ten years of professional experience in fields relevant to One Health, including but not limited to human, animal, or environmental health; (ii) substantial involvement in One Health-related policy formulation, program implementation, or intersectoral collaboration; and (iii) willingness to provide informed consent for participation. For further details, please refer to Appendix 1.

Participants were recruited through a combination of snowball sampling and maximum variation sampling, to ensure a broad and diverse range of perspectives. Snowball sampling enabled access to key informants with extensive networks, while maximum variation sampling helped capture institutional diversity and variation across governance levels and sectors. Initial contact was made via email or telephone to explain the study objectives, ensure eligibility, and secure participation. Written informed consent was obtained from all participants.

### 2.4 Data collection

The research is guided by an analytical framework aligned with the Consolidated Criteria for Reporting Qualitative Research (COREQ) (Appendix 2) and the Standards for Reporting Qualitative Research (SRQR), ensuring methodological rigor. Data were collected through semi-structured in-depth interviews (IDIs) and focus group discussions (FGDs), supplemented by policy document reviews. Semi-structured interview guides were adapted from those developed in the foundational study by Li et al., and were further refined to address the specific objectives of this research. The guides were tailored to different stakeholder groups and pre-tested through a pilot interview, which informed adjustments to question wording and sequencing.

Interviews were conducted between January and April 2024 by trained researchers in Mandarin Chinese. Each interview lasted approximately 50 to 60 min. Interviews with national-level experts were conducted online via Tencent Meeting, while interviews with provincial and local-level participants were conducted in person in a private and secure setting to ensure confidentiality. To establish rapport and encourage openness, the research team explained the study's context and reassured participants of anonymity and data protection at the outset of each session. All interviews were audio-visually recorded with participants'consent and transcribed verbatim. Each transcript was reviewed and cross-validated by a second researcher to ensure accuracy and consistency. Data collection concluded when thematic saturation was reached, determined by the research team's consensus that no new concepts were emerging from subsequent interviews.

### 2.5 Data analysis

The analysis followed a grounded theory approach, incorporating iterative coding and thematic analysis. This approach facilitates the extraction of themes related to governance strategies, challenges, and potential solutions. Two professionally trained researchers independently coded all interview transcripts using NVivo 12 qualitative analysis software. A weekly calibration meeting system was implemented, wherein coding discrepancies were resolved through consensus discussions, achieving a final intercoder agreement rate of 95 %. The process involved three phases:

(i) Open coding: Two researchers (initials blinded for review) independently coded transcripts line-by-line to identify initial concepts, using both inductive (data-driven) and deductive (framework-informed) codes. Discrepancies were resolved through consensus discussions.
(ii) Axial coding: Codes were grouped into categories (e.g., “barriers to intersectoral collaboration,” “enablers of policy integration”) and mapped to the study's conceptual framework. Relationships between categories were examined to identify overarching themes.
(iii) Selective coding: A core narrative was synthesized, integrating key themes into a coherent analytical structure. Member checking was performed with all participants to validate interpretations.

A consensus-driven codebook (Appendix 3) is developed to provide clarity and consistency in categorizing data. Based on grounded theory and a rigorous coding process, we developed the analytical framework presented in Table 1, which identifies the core domains essential to One Health implementation—Monitoring and Evaluation, Intervention and Response, Surveillance and Early Warning, and Capacity Building—as well as key entry points for operationalizing One Health, including Human Resources, Technology, Information, and Financing. The final analysis adhered to COREQ guidelines, with results presented as thematic narratives supported by illustrative quotes.

**Table 1:** Analytical framework of One Health implementation and entry points in China.

Category	Domain	Description
Implementation scheme	Monitoring & evaluation	Develops frameworks to assess effectiveness, ensure accountability, and incorporate feedback for continuous improvement.
	Intervention & response	Enhances coordination across sectors for timely emergency responses, disease control, and public health preparedness.
	Surveillance & early warning	Implements integrated surveillance systems for early detection of health risks across human, animal, and environmental sectors, enabling informed decision-making.
	Capacity building	Focuses on training, workforce development, and institutional learning to ensure long-term sustainability of One Health governance.
Operational entry points	Technology	Utilizes data management systems, diagnostic tools, and digital health platforms to improve surveillance, coordination, and response.
	Information	Emphasizes data collection, sharing, and public communication to support decision-making and transparency across sectors.
	Human resources	Develops an interdisciplinary workforce through training programs and promotes collaboration across human, animal, and environmental health sectors.
	Finance	Focuses on resource allocation, financial sustainability, and cost-effectiveness to support long-term One Health initiatives.

## 3 Results

### 3.1 Current situation and gaps in One Health implementation

#### 3.1.1 Monitoring and evaluation system: Deficiencies in real-time dynamic assessment and comprehensive performance metrics

Current evaluation mechanisms mainly rely on periodic reports and upper-level reviews, lacking real-time dynamic assessment. Although Yongxiu County has promoted internal coordination and linked assessment to funding, the absence of a systematic evaluation framework limits timely reflection of One Health implementation effectiveness.

*“One aspect is risk communication, including internal communication where information is reported to city and county personnel and leaders, as well as external and public communication. For example, we often post risk alerts on our website, informing the public about prevalent diseases and encouraging caution. Of course, such communication must be handled skillfully to avoid causing public panic or misinterpretation.”*

*(Participant-6, sub-national level, administrative coordinator, human health/zoonotic disease)*

To address this, a structured evaluation system is needed. Some grassroots units have introduced cross-departmental assessment standards tied to funding and included in broader development evaluations. For instance, Yongxiu integrated schistosomiasis control into its high-quality development assessments, linking outcomes to fund allocation. This clarified responsibilities and motivated implementation, yet the lack of a comprehensive incentive mechanism remains a challenge.

*“Strengthening supervision and assessment: To implement the Healthy Jiangxi Action, schistosomiasis control work has been incorporated into the ‘Healthy Yongxiu Action,’ with specific schistosomiasis prevention content listed as part of the county's high-quality development assessment. The county's Schistosomiasis and Endemic Disease Control Leading Group has set clear assessment standards for the closure of grazing lands and linked assessment results directly to funding for these efforts, effectively promoting schistosomiasis infection source control strategies.”*

*(Report on the Comprehensive Management of Schistosomiasis Control in Yongxiu County)*

#### 3.1.2 Intervention and response mechanism: Coordination gaps in cross-sectoral emergency operations and resource integration

Effective One Health governance relies on strong interdepartmental coordination. In China, systems have been established for joint response and information sharing. For instance, under the Animal Epidemic Prevention Law, health and animal disease departments coordinate during outbreaks like brucellosis. In Yongxiu County, a graded emergency mechanism addresses public health emergencies, while customs use notification systems to report suspected cases. Environmental health governance also involves collaboration—agricultural departments manage livestock operations, and environmental departments handle pollution control. Joint efforts and clear communication between sectors are vital for effective public health protection.

To improve coordination in disease control, establishing a sustainable command center is key. Such a center ensures unified leadership, rapid response, clear responsibilities, and efficient resource allocation during outbreaks. Regions like Yongxiu County have implemented targeted measures based on local needs. For schistosomiasis prevention, the county enforced a ten-year fishing ban on Poyang Lake [6] to reduce human contact with infected waters and cracked down on illegal fishing. The forestry bureau adopted afforestation strategies to suppress snail populations, investing in housing and environmental projects to alter snail habitats and curb disease recurrence [7]. Environmental efforts include enhanced patrols, enforcement, and record-keeping. Wildlife protection has also been strengthened through bans on wild animal consumption and improved market regulation, reducing zoonotic risks. These integrated measures have successfully blocked disease transmission, boosted local incomes, and improved environmental quality.

*“The strict implementation of the ten-year fishing ban on Poyang Lake has forced all fishermen to transition to other industries, preventing contact with epidemic waters and significantly contributing to schistosomiasis prevention... The county public security bureau played a strong deterrent role in the process of promoting the closure of grazing lands, ensuring the safe implementation of the project in the promotion area. During the fishing ban, illegal fishing activities were severely cracked down upon, optimizing the underwater ecological environment and effectively curbing the proliferation and spread of snails.”*

*(Participant-29, local level, administrative coordinator, human health/zoonotic disease)*

In addition, laboratory testing capabilities remain inadequate in responding to emerging pollutants and disease threats. While current capabilities can support routine tasks, challenges remain in advanced areas such as pathogen detection and DNA testing [8]. Rather than simply adding equipment, optimizing laboratory network integration and resource utilisation is key. Priority should be given to enhancing technical expertise and expanding the scope of testing to meet new disease detection needs.

#### 3.1.3 Surveillance and early warning infrastructure: Fragmented data collection systems and delayed risk identification

China's multi-tiered surveillance system combines dynamic monitoring, mathematical modeling, and early warnings through active and passive surveillance. Systems include direct reporting for infectious diseases, animal and wildlife disease surveillance, and environmental monitoring. Despite technical infrastructure, data sharing across departments remains a key challenge. Systems operate in silos, hindering timely information flow. For instance, customs data is difficult to access due to central control and confidentiality, affecting local monitoring effectiveness.

*“Since 2004, a national direct reporting system for infectious diseases has been established. The Infectious Disease Prevention and Control Law stipulates the responsibilities for reporting infectious diseases, including the duties of medical institutions in reporting through media channels. Weekly surveillance of influenza pathogens and severe disease isolations is conducted. If untyped cases are found, we can promptly communicate with the national authorities and send samples for national testing.”*

*(Participant-6, sub-national level, administrative coordinator, human health/zoonotic disease)*

*“Hainan Province is building a ‘multi-point early warning’ system for infectious diseases, which may require us to provide some data. However, customs is centrally managed and not under Hainan Province's jurisdiction, so there may be difficulties in data provision. We can provide some local data, but some data cannot be provided due to confidentiality or restrictions by the customs bureau. These issues need to be resolved through communication.”*

*(Participant-2, sub-national level, administrative coordinator, human health)*

#### 3.1.4 Capacity building programs: Insufficient professional training and workforce development for integrated health management

From provincial to county levels, various trainings have been conducted to raise One Health awareness. Some units promote internal knowledge exchange and skill improvement. Topics range from policy interpretation to wildlife monitoring, strengthening technical and management capacity. While joint large-scale trainings are limited, targeted sessions and expert exchanges have helped improve workforce preparedness. However, talent development remains insufficient, particularly in supporting integrated project implementation.

*“Each year since 2015, Nanchang City has held a fixed closure and grazing ban training class, which includes leaders from various townships, directors of the grazing ban offices, and professional technical personnel from county-level schistosomiasis prevention stations. These sessions may include schistosomiasis prevention promotion and training on detection, diagnosis, treatment, and reporting of schistosomiasis for medical personnel from sentinel hospitals and township health centers in epidemic areas. This training improves the diagnostic and laboratory skills of rural doctors and enhances the prevention and control capabilities of the county, township, and village-level schistosomiasis teams, ensuring the standardized and orderly conduct of schistosomiasis prevention work in our region.”*

*(Participant-30, local level, policy maker, human health)*

### 3.2 Current situation and gaps in One Health entry points

#### 3.2.1 Technological support system: Insufficient diagnostic capacity at grassroots level and regional imbalance in technical resource allocation

China's grassroots health units face critical technological shortcomings, particularly in underdeveloped regions. Several participants noted that the absence of advanced diagnostic tools—such as high-resolution microscopes and real-time PCR machines—has delayed pathogen detection and weakened early response efforts. One interviewee stated: “We don't even have basic PCR equipment in many county-level labs, which means we can't identify pathogens in time.” While provincial institutions were frequently described as regularly updating their technology, our data suggest that many county-level facilities continue to lag significantly behind.

Rapid deployment of new technologies, like molecular diagnostics, is often underutilized due to insufficient training and lack of integration into daily practice. Existing training programs are fragmented and fail to ensure sustained proficiency among local health workers.

High costs further hinder the use of advanced tools at the grassroots level. While cheaper alternatives exist, their recurring costs remain burdensome. Sustained One Health implementation will require greater national investment, cost-effective technology development, and financial subsidies to support diagnostics in economically disadvantaged regions.

*“Our updates are slower, especially at the county level compared to the provincial level, including our laboratory service capabilities, which we still can't keep up with.”*

*(Participant-31, local level, administrative coordinator, animal health/environment health)*

#### 3.2.2 Information sharing mechanism: Barriers to cross-sectoral data interoperability and absence of real-time monitoring systems

Despite some progress in interdepartmental communication, data sharing across human, animal, and environmental sectors remains fragmented. Most exchanges rely on informal channels like phone calls or joint meetings, lacking a centralized, real-time platform.

Several participants noted that data-sharing delays, inconsistent formats, and confidentiality restrictions have hindered information integration and reduced the effectiveness of coordinated responses. One interviewee remarked: “Different departments use their own systems, and we can't access each other's data in real time.” Drawing from these accounts, we argue that such fragmentation severely limits cross-sector collaboration.

*“In terms of systems, there are still barriers to data sharing. For example, when we initially tried to obtain monitoring data from the agricultural department, they had concerns and their own regulations. If they were to share data with us, it would require approval from higher authorities. The same applies to sharing our data with them. The existing direct reporting system primarily covers medical institutions, and animal data is not included in our system—they have their own system, which is also national. Therefore, data sharing still needs further breakthroughs.”*

*(Participant-6, sub-national level, administrative coordinator, human health/zoonotic disease)*

#### 3.2.3 Human resource development: Structural shortage of professionals and discontinuity in grassroots service capabilities

Grassroots public health systems suffer from an aging workforce, high turnover, and a severe shortage of multidisciplinary professionals. Limited funding and uncompetitive salaries hinder recruitment and retention, especially in fields like veterinary medicine and environmental health. Many trained personnel leave for better-paying positions elsewhere, creating gaps in diagnostic and surveillance capacity. Short-term training programs fail to address long-term workforce needs or equip staff to handle complex One Health challenges. Sustainable workforce development requires increased financial support, clear career pathways, and long-term training strategies tailored to the practical demands.

*“The Forestry Bureau had 10 retirements last year and none hired. Who will do the work of these 10 people? Another 10 will retire this year, and no new hires have been made. The aging problem is severe. Who will be trained? Training is not something that can be accomplished overnight, and even after training, they still may not be proficient.”*

*(Participant-28, local level, administrative coordinator, animal health)*

#### 3.2.4 Financial safeguard mechanism: Project-based funding model and shortage of sustainable operational funds

China has significantly increased funding for disease control programs targeting schistosomiasis and brucellosis, reflecting strong central commitment to One Health. However, this funding often focuses on statutory projects, with limited support for operational needs or innovation. Many local governments adopt a phased approach to project implementation due to budget constraints, prioritizing certain interventions at the expense of comprehensive strategies. The reliance on central-local financial matching also creates regional disparities when local funds are insufficient.

To ensure long-term sustainability, enhanced budget planning, improved central-local coordination, and diversified funding—including public-private partnerships—are needed to support the broader goals of One Health governance.

*“First, the county finance has increased funding for schistosomiasis control year by year, with a total investment of 4.3 million yuan over the past five years dedicated to schistosomiasis control work.”*

*(Participant-27, local level, policy maker, human health)*

*“The government only funds statutory projects, such as infectious disease laboratories for entry and exit personnel. Other projects require laboratories to solve funding issues on their own. Due to limited income, it's difficult for laboratories to purchase equipment, further affecting efficiency and performance.”*

*(Participant-2, sub-national level, administrative coordinator, human health)*

A summary of the current progress, key challenges, and policy recommendations for each component of the One Health governance framework in China is provided in Table 2. The complete version is available in the Appendix 4.

**Table 2:** Overview of One Health governance in China: Progress, challenges, and recommendations.

One Health framework	Current progress	Key challenges	Policy suggestions
*One Health governance implementation*			
Monitoring & evaluation	Authorities oversee periodic reporting on epidemics and human resources.	Absence of real-time evaluation hinders One Health effectiveness.	Adopt a localized the Global One Health Index (GOHI) framework for real-time One Health assessment.
	Yongxiu County links schistosomiasis control to development goals, with performance-based funding.	Low incentives weaken One Health enforcement.	Tie incentives to One Health targets to ensure accountability.
Intervention & response	Tiered emergency response system improves interdepartmental coordination.	Communication inefficiencies delay joint responses.	Establish centralized Emergency Response Coordination Office.
	Fishing bans and ecological projects show successful management outcomes.	Resource allocation and enforcement need improvement.	Form National Resource Allocation and Enforcement Committee.
Surveillance & early warning	Three-tier disease surveillance enables timely risk assessment.	Poor inter-departmental coordination and data integration.	Create Unified Data Integration Platform for better data sharing.
	Surveillance covers animal health, food safety, and environment.	Lack of cross-departmental data sharing mechanisms.	Implement National Data Sharing Framework for integrated monitoring.
	Combined active/passive monitoring enables quick risk identification.	Delays in monitoring reduce warning effectiveness.	Enhance system with real-time reporting technologies.
Capacity building	A comprehensive capacity-building training program, spanning from provincial to grassroots units, enhances the ability to address public health challenges effectively.	Existing training programs lack large-scale joint sessions and continuous skill enhancement.	Establish a National Training Institute for joint training and continuous professional development to meet evolving workforce needs.

*One Health governance entry points*			
Human resource	Talent strategies improving grassroots public health appeal	Implementation gaps in salaries, career development, and infrastructure hinder recruitment/retention	Implement comprehensive incentive package: - Competitive salaries & bonuses - Clear career paths & training - Improved facility investments
	Multidisciplinary teamwork models enhancing collaboration	Severe shortage of diverse professionals limits service quality	Launch targeted recruitment/training program with competitive packages & continuous development
Technology	Government investing in technical equipment upgrades	Regional disparities in diagnostic capabilities delay treatment	Prioritize equipment distribution to underserved areas with staff training programs
	Some regions implementing tech training programs to improve tool proficiency	Grassroots tech updates lag behind cities; training not keeping pace with advancements	Establish continuous adaptive training with: - Regular hands-on sessions - Centralized online learning platform
Information	Basic inter-department communication exists (reports, calls, meetings)	Data sharing hindered by: - Confidentiality concerns - Inconsistent standards - System incompatibility	Create secure data-sharing framework with: - Unified standards - Quality protocols - Encryption - Joint training workshops
	Departments recognize data sharing importance for One Health	Technical/policy barriers exist for sensitive data sharing	Establish clear guidelines for: - Data handling standards - Secure interdepartmental exchange - Oversight committee
	Some institutionalized sharing exists (regular reports, anomaly alerts)	Inefficient emergency information sharing	Create AI-powered emergency data platform + conduct regular drills
Finance	Increasing local funding for disease control (special infectious disease funds)	Funding covers only project costs, not personnel allowances	Expand funding to include: - Personnel allowances - Comprehensive incentive framework
	Central-local financial coordination enables timely funding		Revise allocation guidelines to cover both projects and personnel

## 4 Discussion

The integration of One Health governance in China represents a comprehensive effort to address interconnected health risks at the human-animal-environment interface. However, the current state of implementation reveals both promising initiatives and significant challenges, which must be addressed to enhance sustainability and effectiveness across the governance framework. While monitoring and evaluation mechanisms exist, their current limitations hinder the ability to capture the dynamic and complex nature of zoonotic disease risks and public health threats. As it stands, the reliance on periodic reporting means real-time insights are lacking, which are essential for timely decision-making [9]. For instance, as seen in the Yongxiu County model, where assessment outcomes are linked to interdepartmental coordination and funding allocation, localized successes—such as in controlling schistosomiasis—have been achieved. However, such models remain fragmented and are insufficiently integrated into a cohesive national One Health framework.

Aligned with existing calls for standardized metrics, tools such as the Global One Health Index (GOHI) [10,11] offer a path forward by facilitating integration across human, animal, and environmental sectors. A national One Health framework based on real-time evaluation could provide the continuous monitoring necessary to refine response strategies and optimize resource allocation. Importantly, aligning evaluation outcomes with funding mechanisms would encourage grassroots adoption of One Health practices, thus improving both effectiveness and accountability at all governance levels.

Building on literature that emphasizes the importance of actionable governance, the shift from monitoring to intervention is critical. Coordinated response systems are vital in addressing zoonotic outbreaks, antimicrobial resistance, and food safety threats [12]. Although China has developed interdepartmental protocols for addressing public health emergencies—such as the Animal Epidemic Prevention Law [5] for zoonotic diseases like brucellosis—full institutionalization of these protocols across all government levels remains a challenge. The experience of global best practices, such as the U.S. Federal Emergency Management Agency (FEMA) [13] and Germany's Federal Office of Civil Protection and Disaster Assistance (BBK), offers valuable lessons. FEMA's centralized command structure ensures that communication and coordination are streamlined across federal, state, and local levels. Similarly, BBK's disaster management framework [14,15], which integrates resource pre-positioning and allocation based on risk assessments, could be adapted to address China's regional disparities in resource distribution, particularly in rural areas.

To enhance intervention readiness, establishing a centralized One Health Command Center in China could improve resource integration and interdepartmental coordination. Such a center would play a crucial role in ensuring that timely and efficient interventions can take place during public health crises. Moreover, adopting pre-positioning strategies based on region-specific risk assessments would help China's system become more adaptable and responsive to emerging threats.

As emphasized in global reviews of surveillance systems, early warning mechanisms are essential for detecting emerging threats. China's current multi-tiered surveillance system provides some valuable tools; however, there is a clear need for further integration and more advanced real-time analytics. Drawing inspiration from global models like the European Union's Joint Research Centre (JRC) [16] and the United Kingdom's NHS Digital platform [17,18], China could integrate data from diverse sources—including health, agricultural, and environmental sectors—to enhance crisis management and risk prediction. JRC's use of data analytics and modeling tools for real-time risk forecasting presents a potential model for China's own surveillance system. By adopting similar methods, China could create a unified national platform that consolidates data from regional and national levels for more cohesive monitoring and risk assessment. This would not only improve early warning system efficiency but also promote greater cross-sectoral collaboration—a central pillar of One Health governance.

Capacity building has been consistently cited as foundational to One Health implementation. Although there have been notable improvements at the provincial and municipal levels, rural areas continue to face significant shortages in trained personnel and diagnostic resources. Limited access to advanced diagnostic tools delays zoonotic disease detection. Following models like Singapore's Civil Service College (CSC) [19], China could establish a centralized institution focused on One Health training. This would standardize training across sectors, helping public servants tackle complex health challenges more effectively. Additionally, adopting Canada's approach to interdisciplinary training—bringing together veterinarians, environmental scientists, and public health professionals [20]—would foster a more holistic understanding of One Health within China's workforce.

Moreover, investing in diagnostic equipment, mobile health units, and laboratory capacity is crucial for improving surveillance and response in remote areas. Additionally, targeted training programs for healthcare workers in underserved regions, alongside investments in portable technologies like Clustered Regularly Interspaced Short Palindromic Repeats (CRISPR)-based diagnostic tools, could help bridge the urban-rural divide and enhance the ability to respond to emerging health threats in these areas [21].

In line with structural barrier analyses from prior studies, addressing key bottlenecks in technology, information systems, human resources, and financing is essential. Rural technological gaps hinder timely detection and response, while fragmented data systems and the absence of a standardized platform limit real-time decision-making [22]. Another challenge is the recruitment and retention of skilled professionals, especially in rural areas, where competitive salaries, career development opportunities, and necessary infrastructure are often lacking [23].

Financial limitations—especially under centralized funding models—restrict scalability. Exploring alternative financing models, including public-private partnerships [24], could diversify funding sources and foster innovation. Targeted needs assessments and efficient use of existing equipment and infrastructure would further enhance programmatic impact.

The sustainability of China's One Health framework will ultimately depend on overcoming these structural and operational challenges [25]. Strengthening monitoring and evaluation systems, enhancing intervention coordination, integrating surveillance platforms, and investing in capacity building—especially at the grassroots level—are all crucial to creating a more resilient and effective One Health governance model. Addressing the key barriers in technology, information sharing, human resources, and finance will also ensure that the One Health framework remains adaptable and scalable in the face of emerging health threats.

Despite its contributions to understanding One Health governance in the Chinese context, this study has certain limitations inherent to its design and scope. As a qualitative inquiry focused on expert perspectives, the findings are shaped by the positionality and institutional affiliations of selected participants. While purposive sampling ensured a level of sectoral and geographic diversity, the voices of frontline personnel, civil society actors, and communities directly affected by One Health-related challenges remain less visible. Their lived experiences and operational insights—often crucial for assessing policy effectiveness on the ground—may therefore be underrepresented.

Furthermore, the study's temporal and geographic scope was necessarily constrained by logistical considerations. The selection of two provinces for fieldwork provides contextual depth but limits the generalizability of observations across China's highly heterogeneous administrative and ecological settings. Governance structures, intersectoral coordination capacity, and disease burden can vary significantly between regions, meaning that insights from one locality may not reflect dynamics elsewhere. In addition, this research is situated within a specific moment of policy evolution. One Health governance is rapidly developing, both conceptually and institutionally, in China and globally. As such, the study captures an important but transitional phase, and may not fully anticipate emergent models, decentralized adaptations, or unformalized mechanisms that evolve in response to new public health and environmental pressures.

Future research could benefit from incorporating community-level ethnographic methods, comparative subnational case studies, and quantitative evaluations of governance performance. Such approaches would complement expert narratives and enrich understanding of how One Health principles are translated into action across different administrative tiers and socio-ecological contexts.

## 5 Conclusion

As China continues to refine its One Health governance model, international best practices offer valuable reference points. Nevertheless, the primary focus should be on developing a system tailored to China's specific context—one that effectively addresses the nation's regional disparities, resource limitations, and distinct governance structures. Building such a customized framework can help tackle domestic public health challenges and improve cross-sectoral cooperation, data integration, and resource allocation.

This study identifies critical shortcomings in real-time monitoring, cross-sectoral coordination, data sharing, workforce capacity, and financial sustainability. Tackling these issues is essential for building a resilient and effective One Health governance framework in China. The results not only offer evidence-based guidance for enhancing domestic governance but also provide useful reference for other countries confronting comparable multisectoral challenges at the human–animal–environment interface.

## CRediT authorship contribution statement

**Qi-yu Zhang:** Writing – review & editing, Writing – original draft, Visualization, Software, Resources, Project administration, Methodology, Investigation, Formal analysis, Data curation, Conceptualization. **Yan-Yan Zhang:** Writing – review & editing, Writing – original draft, Visualization, Software, Resources, Project administration, Methodology, Investigation, Formal analysis, Data curation, Conceptualization. **Jing-Shu Liu:** Writing – review & editing, Writing – original draft, Visualization, Software, Resources, Project administration, Methodology, Investigation, Formal analysis, Data curation, Conceptualization. **Xin-Chen Li:** Writing – review & editing, Writing – original draft, Visualization, Software, Resources, Project administration, Methodology, Investigation, Formal analysis, Data curation, Conceptualization. **Ze-Lin Zhu:** Writing – review & editing, Investigation. **Xin-Yu Feng:** Writing – review & editing, Investigation. **Le-Fei Han:** Writing – review & editing, Investigation. **Chen-Di Zhu:** Writing – review & editing. **Hein-Min Tun:** Writing – review & editing. **Lian Zong:** Writing – review & editing, Writing – original draft, Methodology, Conceptualization. **Xiaoxi Zhang:** Writing – review & editing, Writing – original draft, Supervision, Methodology, Investigation, Conceptualization.

## Consent for publication

Not applicable.

## Ethics approval and consent to participate

The study protocol was reviewed and approved by the Ethics Committee of Public Health and Nursing Research, Shanghai Jiao Tong University School of Medicine (SJUPN-2024-036-KS1).

## Fundings

The project was supported by 10.13039/501100001809National Natural Science Foundation of China Young Scientists Fund (No. 72204160), Shanghai Municipal Health Commission Clinical Research Special Fund for the Healthcare Industry (No. 20244Y0007) and Three-Year Initiative Plan for Strengthening Public Health System Construction in Shanghai (2023–2025) Key Discipline Project (No. GWVI-11.1-12).

## Declaration of competing interest

The authors declare that they have no competing interests.

## Data Availability

The full study protocol and the datasets, are available, following manuscript publication, upon request from the corresponding author (Xiao-Xi Zhang, zhangxiaoxi@sjtu.edu.cn).

## Appendix A Supplementary data

Supplementary material 1 Participants Information Supplementary material 1

Supplementary material 2 COREQ Checklist Supplementary material 2

Supplementary material 3 Codebook Supplementary material 3

Supplementary material 4 Overview of One Health Governance in China: Progress, Challenges, and Recommendations Supplementary material 4

## References

[1] FAO O (2008). https://www.fao.org/4/aj137e/aj137e00.htm.

[2] Li O.Y., Wang X., Yang K., Liu D., Shi H. (2023). The approaching pilot for One Health governance index. Infect. Dis. Poverty.

[3] Rüegg S.R., Häsler B., Zinsstag J. (2018).

[4] Li X., Zhang Y., Zhang Q., Liu J., Zhu Z., Feng X. (2024). Strategy and mechanism of One Health governance: case study of China. Sci. One Health..

[5] (2021). Animal epidemic prevention law of the People's Republic of China. Animal Husbandry Industry..

[6] Zeng S. (2021). “Ten-year fishing ban” protects the Yangtze River. Rural Work Communications..

[7] (2022). Technical Regulations for Improving the Effectiveness of Forestry Schistosomiasis Control and Snail Suppression, LY/T 3333–2022.

[8] Che Y., Sun Q., Wang X. (2023). Current status, problems, and countermeasures of testing capabilities in primary veterinary laboratories. Shandong J. Animal Sci. Vet. Med..

[9] Leifels M., Khalilur Rahman O., Sam I.C., Cheng D., Chua F.J.D., Nainani D. (2022). The One Health perspective to improve environmental surveillance of zoonotic viruses: lessons from COVID-19 and outlook beyond. ISME Commun..

[10] Zhang X.X., Liu J.S., Han L.F., Xia S., Li S.Z., Li O.Y. (2022). Towards a global One Health index: a potential assessment tool for One Health performance. Infect. Dis. Poverty.

[11] Zhang Q., Liu J., Han L., Li X., Zhang C., Guo Z. (2024). How far has the globe gone in achieving One Health? Current evidence and policy implications based on global One Health index. Sci. One Health..

[12] Mackenzie J.S., Mckinnon M., Jeggo M. (2014).

[13] Agency tFEM (2024-12-02). National Incident Management System. https://www.fema.gov/emergency-managers/nims.

[14] Community FMotIa (2024-12-02). The Federal Office of Civil Protection and Disaster Assistance (BBK). https://www.bmi.bund.de/EN/topics/civil-protection/bbk/bbk-node.html.

[15] Wuppertal B. (2024-12-02). Academy for crisis management, Emergency Planning and Civil Protection (AKNZ).

[16] Commission E (2024-12-02). European Crisis Management Laboratory. https://joint-research-centre.ec.europa.eu/laboratories-z/european-crisis-management-laboratory_en.

[17] England N. (2024-12-02). How We Are Developing the Platform. https://digital.nhs.uk/about-nhs-digital/corporate-information-and-documents/national-digital-channels---platform-and-integration-strategy/how-we-are-developing-the-platform.

[18] Faulkner-Gurstein R., Wyatt D. (2023). Platform NHS: reconfiguring a public service in the age of digital capitalism. Sci. Technol. Hum. Values.

[19] College C.S. (2024-12-02). Civil Service CollegeSingapore.

[20] Kelly P. (2022). One Health programs at Canadian universities with a veterinary college-1. The University of Guelph. Can. Vet. J. Revue Veterinaire Canadienne..

[21] McLaren Z.M., Sharp A., Hessburg J.P., Sarvestani A.S., Parker E., Akazili J. (2017). Cost effectiveness of medical devices to diagnose pre-eclampsia in low-resource settings. Dev. Eng..

[22] Tariq M.U. (2025). Generative AI Techniques for Sustainability in Healthcare Security.

[23] Wang L., Wang Z., Ma Q., Fang G., Yang J. (2019). The development and reform of public health in China from 1949 to 2019. Glob. Health.

[24] de Ferranti D., Griffin C.C., Escobar M.-L., Glassman A., Lagomarsino G. (2008). Brookings Global Health Financing Initiative Working Paper.

[25] Huang L., He J., Zhang C., Liu J., Guo Z., Lv S. (2023). China’s One Health governance system: the framework and its application. Sci One Health.

