# Supplementary material for: Integrating One Health governance in China: Assessing structural implementation and operational entry points
Source: One Health. 2025 Sep 17;21:101209. doi: 10.1016/j.onehlt.2025.101209 (PMC12495039; doi:10.1016/j.onehlt.2025.101209)
Supplement: Supplementary material 1 — Participants Information [file mmc1.docx]

**Appendix 1 Participants Information**

| **No.** | **Interview time** | **Interviewee type** | **Working level** | **Working area** |
| --- | --- | --- | --- | --- |
| 1 | 2024/3/11 | administrative coordinator | sub-national level | zoonotic disease |
| 2 | 2024/3/11 | administrative coordinator | sub-national level | human health |
| 3 | 2024/3/11 | policy maker | sub-national level | human health |
| 4 | 2024/3/11 | administrative coordinator | sub-national level | environment health |
| 5 | 2024/3/11 | administrative coordinator | sub-national level | AMR |
| 6 | 2024/3/11 | administrative coordinator | sub-national level | human health/zoonotic disease |
| 7 | 2024/3/12 | administrative coordinator | sub-national level | human health/zoonotic disease |
| 8 | 2024/3/12 | administrative coordinator | sub-national level | environment health |
| 9 | 2024/3/13 | administrative coordinator | local level | human health/zoonotic disease |
| 10 | 2024/3/13 | policy maker | local level | human health |
| 11 | 2024/3/13 | administrative coordinator | local level | environment health |
| 12 | 2024/3/13 | administrative coordinator | local level | human health |
| 13 | 2024/3/13 | administrative coordinator | local level | animal health |
| 14 | 2024/3/13 | administrative coordinator | local level | animal health |
| 15 | 2024/3/13 | policy maker | local level | human health |
| 16 | 2024/3/13 | administrative coordinator | local level | human health/zoonotic disease |
| 17 | 2024/3/13 | administrative coordinator | local level | human health |
| 18 | 2024/3/13 | administrative coordinator | local level | human health |
| 19 | 2024/3/13 | policy maker | local level | animal health |
| 20 | 2024/3/13 | administrative coordinator | local level | animal health |
| 21 | 2024/3/13 | administrative coordinator | local level | environment health |
| 22 | 2024/3/13 | administrative coordinator | local level | environment health |
| 23 | 2024/3/13 | administrative coordinator | local level | environment health |
| 24 | 2024/3/13 | administrative coordinator | local level | human health |
| 25 | 2024/3/13 | administrative coordinator | local level | human health |
| 26 | 2024/3/13 | administrative coordinator | local level | human health |
| 27 | 2024/4/2 | policy maker | local level | human health |
| 28 | 2024/4/2 | administrative coordinator | local level | animal health |
| 29 | 2024/4/2 | administrative coordinator | local level | human health/zoonotic disease |
| 30 | 2024/4/2 | policy maker | local level | human health |
| 31 | 2024/4/2 | administrative coordinator | local level | animal health/environment health/ |
| 32 | 2024/4/2 | administrative coordinator | local level | animal health |
| 33 | 2024/4/8 | technical expert | national level | zoonotic disease |
| 34 | 2024/4/8 | technical expert | national level | animal health/zoonotic disease |
| 35 | 2024/4/8 | technical expert | national level | human health/zoonotic disease |
| 36 | 2024/4/9 | technical expert | national level | zoonotic disease |
| 37 | 2024/4/9 | technical expert | national level | environment health |
| 38 | 2024/4/9 | technical expert | national level | animal health/Food safety |
| 39 | 2024/4/10 | policy maker | local level | animal health |
| 40 | 2024/4/10 | policy maker | local level | human health |
| 41 | 2024/4/10 | policy maker | local level | zoonotic disease/human health |
