# Supplementary material for: Integrating One Health governance in China: Assessing structural implementation and operational entry points
Source: One Health. 2025 Sep 17;21:101209. doi: 10.1016/j.onehlt.2025.101209 (PMC12495039; doi:10.1016/j.onehlt.2025.101209)
Supplement: Supplementary material 3 — Codebook [file mmc3.docx]

**Appendix 3 Codebook**

| **One Health Implementation** | |
| --- | --- |
| 1. Monitoring and  Evaluation | Definition: Monitoring and evaluation are critical processes for assessing the effectiveness and impact of public health programs and interventions. Monitoring involves the ongoing collection of data to track the progress of activities and outcomes, while evaluation is the systematic assessment of the program's performance, including its effectiveness, efficiency, relevance, and sustainability. |
| 2. Intervention and Response | Definition: Intervention and response refer to the coordinated actions taken to mitigate the impact of a health threat once it is identified. This includes immediate measures to control the spread of the disease or outbreak, the allocation of resources, the implementation of preventive actions, and the treatment of affected populations. The effectiveness of interventions depends on timely and appropriate responses based on scientific evidence, local capacities, and the nature of the threat. |
| 3. Surveillance and Early Warning | Definition: Surveillance and early warning systems involve the continuous monitoring of health threats, such as infectious diseases, and the identification of trends or anomalies that could indicate an impending outbreak. Early warning mechanisms rely on the systematic collection, analysis, and dissemination of data from various sources, enabling authorities to detect potential risks early and trigger preventive actions before a public health crisis occurs. |
| 4. Capacity-building | Definition: Capacity-building refers to the process of strengthening the abilities and resources of individuals, organizations, and systems to effectively manage public health risks. This includes improving knowledge, skills, infrastructure, and governance to enhance preparedness, response, and recovery capabilities. Capacity-building may focus on training health professionals, equipping health facilities, or enhancing the overall public health infrastructure. |
| **One Health Entry points** | |
| 1. Human resources | Definition: It refers to the number and structure of people involved in the field, covering professionals from multiple disciplinary backgrounds such as medicine, veterinary medicine, environmental science, epidemiology and policy development. It focuses on the objective description of human resources, including the distribution of their areas of specialisation, sectoral affiliation and geographical distribution. |
| 2. Technology | Definition: It refers to the specific technological tools and methods required, including but not limited to disease diagnostic equipment, data collection and processing software, and outbreak surveillance systems. |
| 3. Information | Definition: It refers to the data and knowledge generated, processed and applied by One Health and its scope, including disease surveillance data, environmental monitoring data, policy documents, etc. |
| 4. Financing | Definition: It relates to the source, size and distribution of funds, including project budgets, sources of funding (e.g. government budgets, funding from international organisations, private donations, etc.), and the specific allocation and use of funds. |
