# Supplementary material for: Integrating One Health governance in China: Assessing structural implementation and operational entry points
Source: One Health. 2025 Sep 17;21:101209. doi: 10.1016/j.onehlt.2025.101209 (PMC12495039; doi:10.1016/j.onehlt.2025.101209)
Supplement: Supplementary material 4 — Overview of One Health Governance in China: Progress, Challenges, and Recommendations [file mmc4.docx]

**Appendix 4 Overview of One Health Governance in China: Progress, Challenges, and Recommendations**

| One Health Framework | Current Progress | Key Challenges | Policy Suggestions |
| --- | --- | --- | --- |
| One Health Governance Implementation | | | |
| Monitoring & Evaluation | Periodic reporting mechanisms ensure regular updates on epidemics and human resources, overseen by higher authorities. | Lack of a real-time evaluation system and systematic framework limits assessment of One Health effectiveness. | Establish a real-time One Health evaluation system by localizing the Global One Health Index (GOHI) framework to enhance responsiveness and adaptability. |
|  | Yongxiu County integrates schistosomiasis control into its high-quality development assessments, allocating funds based on performance to advance One Health initiatives. | Insufficient incentives result in weak enforcement of One Health measures. | Implement a performance management framework linking incentives to One Health targets, ensuring accountability and motivation. |
| Intervention& Response | A tiered emergency response system facilitates effective interdepartmental communication and cooperation within the government. | Inefficiencies in communication and collaboration delay joint response efforts. | Create a centralized Emergency Response Coordination Office to improve interdepartmental communication and joint response efficiency. |
|  | Intervention measures such as fishing bans, ecological improvement projects, and strict crackdowns on illegal fishing have achieved significant management outcomes. | Resource allocation, enforcement, and sustainability require further improvement. | Form a National Resource Allocation and Enforcement Committee to guarantee equitable resource distribution, policy enforcement, and sustainability. |
| Surveillance & Early warning | A three-tier infectious disease surveillance and early warning system allows for timely disease monitoring and comprehensive risk assessment. | Despite a mature monitoring system, inter-departmental coordination and data integration remain inadequate. | Develop a Unified Data Integration Platform for seamless inter-departmental data sharing, enhancing monitoring effectiveness. |
|  | The surveillance system encompasses animal health, food safety, and environmental quality, ensuring broad coverage of health-related areas. | Data integration challenges persist, needing cross-departmental sharing mechanisms. | Set up a National Data Sharing Framework to integrate cross-sectoral monitoring data, improving decision-making accuracy. |
|  | By combining active and passive monitoring, the system can promptly identify potential risks and swiftly respond to emergencies. | Delays in active and passive monitoring hinder timely warnings. | Strengthen the monitoring system with real-time reporting technologies and protocols to reduce delays and omissions. |
| Capacity Building | A comprehensive capacity-building training program, spanning from provincial to grassroots units, enhances the ability to address public health challenges effectively. | Existing training programs lack large-scale joint sessions and continuous skill enhancement. | Establish a National Training Institute for joint training and continuous professional development to meet evolving workforce needs. |
| One Health Governance Entry Points | | | |
| Human Resource | Policies are beginning to emphasize and improve talent strategies to enhance the appeal of the grassroots public health system. Despite policy awareness, actual implementation lags in areas such as salary levels, career development opportunities, and infrastructure improvements, affecting talent recruitment and retention. | Despite policy awareness, actual implementation lags in areas such as salary levels, career development opportunities, and infrastructure improvements, affecting talent recruitment and retention. | To effectively address the challenges faced by the grassroots public health system in talent recruitment and retention, it is recommended to implement a comprehensive incentive policy. This policy should include increasing the salary levels of grassroots public health workers, providing clear career development paths, and enhancing investment in infrastructure. Specific measures could include establishing special funds, raising the basic salaries of grassroots medical staff, and offering additional performance bonuses; creating a systematic career advancement mechanism, offering more training and continuing education opportunities; and increasing investment in the hardware facilities of grassroots health institutions to improve the working environment. |
|  | Promote multidisciplinary team work models, enhance collaboration among professionals with different backgrounds, and improve the overall efficiency and effectiveness of public health efforts. | There is a severe shortage of multidisciplinary talents at the city, county, and township levels, particularly in the grassroots public health sector, which limits the quality and scope of services. | To address the severe shortage of multidisciplinary talents at the city, county, and township levels in the grassroots public health sector, it is recommended to establish a targeted recruitment and training program. This program should focus on attracting and developing professionals from various disciplines, offering competitive salaries, scholarships, and continuous professional development opportunities. By fostering a diverse and skilled workforce, the program will enhance collaboration and improve the overall efficiency and effectiveness of public health efforts. |
| Technology | The government has started investing in updating technical equipment for grassroots health systems to enhance the diagnostic capabilities for local diseases. | There is a significant disparity in technical capabilities across regions, with grassroots units particularly lacking advanced diagnostic equipment, impacting timely diagnosis and effective treatment. | To address the significant disparity in technical capabilities across regions and enhance the diagnostic capabilities of grassroots health systems, it is recommended to implement a targeted investment and resource allocation program. This program should prioritize the distribution of advanced diagnostic equipment to underserved regions, ensuring equitable access to necessary tools for timely diagnosis and effective treatment. Additionally, providing ongoing technical training for local healthcare professionals will maximize the utilization of new equipment. |
|  | With the introduction of new technologies, some regions have started implementing adaptive training programs to help technical personnel master new tools proficiently, thus enhancing the efficiency of technological applications. | The update of grassroots technology lags behind provincial and municipal levels, and training programs fall behind technological advancements, hindering the improvement of grassroots technical capabilities. | To address the lag in technology updates and the inadequacy of training programs at the grassroots level, it is recommended to establish a continuous and adaptive training initiative that aligns with the pace of technological advancements. This initiative should include regular updates and hands-on training sessions for technical personnel, ensuring they remain proficient with the latest tools and technologies. Additionally, creating a centralized online platform for training resources and real-time support can facilitate ongoing education and skill enhancement. |
| Information | A basic information communication mechanism exists across departments, initially achieving information exchange through work reports, phone calls, document sharing, and joint meetings. | The establishment of an information-sharing platform is hindered by data confidentiality between departments, differing data standards, variations in data quality, and the lack of interoperability between information systems. | To overcome the barriers to establishing an effective information-sharing platform, it is recommended to develop a standardized, secure data-sharing framework that addresses confidentiality concerns and ensures interoperability between departmental information systems. This framework should include uniform data standards, protocols for data quality assurance, and robust encryption methods to protect sensitive information. Additionally, fostering inter-departmental collaboration through regular joint training sessions and workshops will promote consistent data practices and enhance mutual understanding. |
|  | Currently, all departments hold a positive attitude towards data sharing, recognizing its importance in strengthening the "One Health" framework. | Data sharing faces both technical and policy barriers in practice, with a lack of clear policy guidance on handling sensitive data and interdepartmental data sharing. | To facilitate effective data sharing within the "One Health" framework,it is recommended to establish clear policy guidelines that address both technical and policy barriers. These guidelines should outline standardized procedures for handling sensitive data, ensure compliance with data protection regulations, and promote secure interdepartmental data exchange. Additionally, the creation of a dedicated oversight committee to monitor and enforce these guidelines will help maintain data integrity and foster trust among departments. |
|  | Information sharing has been institutionalized to some extent, for example, through regular data reporting and a rapid reporting system for anomalies. | This institutionalized implementation faces challenges of insufficient efficiency and effectiveness, with obstacles remaining in achieving rapid and comprehensive information sharing during emergencies. | To enhance the efficiency and effectiveness of institutionalized information sharing, it is recommended to develop an integrated emergency response data platform that enables real-time data exchange and analysis. This platform should leverage advanced technologies such as artificial intelligence and machine learning to rapidly identify and disseminate critical information during emergencies. Additionally, regular simulation exercises and drills should be conducted to ensure all departments are proficient in using the platform. |
| Finance | Local governments have shown a trend of increasing financial support for disease prevention and control efforts annually, such as establishing special funds for the prevention and control of relevant infectious diseases. | The government only provides funding for statutory projects, and financial support mainly covers project expenses, excluding allowances for project personnel, which further impacts the progress and effectiveness of the work. | To address the limitations in financial support for disease prevention and control efforts, it is recommended to expand the scope of funding to include allowances for project personnel. Establishing a comprehensive funding framework that covers both project expenses and personnel incentives will ensure sustained motivation and efficiency among staff, thereby enhancing the overall effectiveness of disease prevention initiatives. |
|  | The coordination between central transfer payments and local finances ensures timely fund allocation. |  | To improve the effectiveness of financial support  for disease prevention and control, it is recommended to revise the funding allocation guidelines to include allowances for project personnel in addition to project expenses. This will ensure that the workforce is adequately compensated, thereby enhancing their motivation and the overall progress of the projects. |
